# Plant-Based Diets Are Associated With Lower Adiposity Levels Among Hispanic/Latino Adults in the Adventist Multi-Ethnic Nutrition (AMEN) Study

**DOI:** 10.3389/fnut.2019.00034

**Published:** 2019-04-09

**Authors:** Pramil N. Singh, Karen Jaceldo-Siegl, Wendy Shih, Nancy Collado, Lap T. Le, Krystal Silguero, Dennys Estevez, Michael Jordan, Hector Flores, David E. Hayes-Bautista, William J. McCarthy

**Affiliations:** ^1^Center for Nutrition, Healthy Lifestyles and Disease Prevention, School of Public Health, Loma Linda University, Loma Linda, CA, United States; ^2^Center for Health Research, School of Public Health, Loma Linda University, Loma Linda, CA, United States; ^3^Center for Hispanic Health, White Memorial Medical Center, Los Angeles, CA, United States; ^4^Department of Family Medicine, White Memorial Medical Center, Los Angeles, CA, United States; ^5^Center for Study of Latino Health and Culture, David Geffen School of Medicine, University of California at Los Angeles, Los Angeles, CA, United States; ^6^Health Policy and Management, School of Public Health, UCLA Jonsson Comprehensive Cancer Center, Los Angeles, CA, United States

**Keywords:** Hispanic/Latino, plant-based diet, vegetarian, obesity, Seventh-day Adventist

## Abstract

**Background:** The Hispanic/Latino population in the US is experiencing high rates of obesity and cardio-metabolic disease that may be attributable to a nutrition transition away from traditional diets emphasizing whole plant foods. In the US, plant-based diets have been shown to be effective in preventing and controlling obesity and cardio-metabolic disease in large samples of primarily non-Hispanic subjects. Studying this association in US Hispanic/Latinos could inform culturally tailored interventions.

**Objective:** To examine whether the plant-based diet pattern that is frequently followed by Hispanic/Latino Seventh-day Adventists is associated with lower levels of adiposity and adiposity-related biomarkers.

**Methods:** The Adventist Multiethnic Nutrition Study (AMEN) enrolled 74 Seventh-day Adventists from five Hispanic/Latino churches within a 20 mile radius of Loma Linda, CA into a cross-sectional study of diet (24 h recalls, surveys) and health (anthropometrics and biomarkers).

**Results:** Vegetarian diet patterns (Vegan, Lacto-ovo vegetarian, Pesco-vegetarian) were associated with significantly lower BMI (24.5 kg/m^2^ vs. 27.9 kg/m^2^, *p* = 0.006), waist circumference (34.8 in vs. 37.5 in, *p* = 0.01), and fat mass (18.3 kg vs. 23.9 kg, *p* = 0.007), as compared to non-vegetarians. Adiposity was positively associated with pro-inflammatory cytokines (Interleukin-6) in this sample, but adjusting for this effect did not alter the associations with vegetarian diet.

**Conclusions:** Plant-based eating as practiced by US-based Hispanic/Latino Seventh-day Adventists is associated with BMI in the recommended range. Further work is needed to characterize this type of diet for use in obesity-related interventions among Hispanic/Latinos in the US.

## Background

The rates of obesity and diabetes have sharply increased in the last decade among Hispanics/Latinos in North America (1–4). Currently, diabetes is the fifth leading cause of death in Hispanics/Latinos, and the trend represents a health disparity whereby rates are 60% higher among Hispanics/Latinos as compared to non-Hispanic Whites and increasing faster than in other major ethnic groups (3, 5). In a large sample of Hispanic/Latino adults in four regions of United States (*n* = 16,400), the Hispanic Community Health Study/Study of Latinos found that about 1 in 3 individuals had pre-diabetes, and that only about half of individuals with type 2 diabetes (T2D) had it under control (6).

One potential contributor to this burden of obesity and diabetes is the occurrence of a “nutrition transition” occurring in most people of Mexican origin but accelerated in US Hispanic/Latinos as they acculturate to consuming higher intake of meats and processed foods, and lower intake of whole plant foods commonly found in the traditional diet (i.e., squash, maize, beans) (7–9). For example, the analysis of a nationally representative sample of US Mexican adults found higher rates of overweight/obesity in those who had “transitioned” from traditional eating patterns emphasizing maize to patterns emphasizing red meat, processed meat, and processed foods (10–12). These emerging data support that a plant-based dietary intervention could be effective in primary and secondary prevention of obesity and obesity-related diabetes in the Hispanic/Latino population.

Overall, in a systematic review of 15 intervention studies (17 intervention groups among them) in multi-ethnic samples, Barnard et al. has shown that interventions with the vegetarian diet contributed to an average of more than 3 kg of weight loss (13). For diabetics, a similar systematic review of vegetarian diet interventions indicated significant improvement in glycemic control (14).

A recent pilot study at Loma Linda University has shown that a 5-week plant-based, culturally-tailored diet intervention implemented through community clinics and a church significantly improved hemoglobin A1C levels over a 6 month follow-up in 32 Latino diabetics living in a medically underserved area. At Loma Linda University, more than 60 years of NIH-funded prospective cohort studies [1960 Adventist Mortality Study (15), 1976 Adventist Health Study-1 (16), 2002 Adventist Health Study-2 (17)] have shown that the vegetarian diet was associated with lower risk of weight gain (18, 19), stroke (20), and diabetes (21, 22), and on this causal pathway, a longer life expectancy. These cohorts enrolled Seventh-day Adventists (most recently a nationwide cohort of 96,000 multi-ethnic adults in AHS-2) who provide a unique insight into diet since, due to faith-based recommendations, about 50% are vegetarian, and virtually all avoid smoking and alcohol (23). Faith-based counsels on diet also encourage the consumption of specific plant foods (i.e., legumes, nuts) in place of animal products (19). Hypotheses generated from these cohorts have been tested in landmark intervention trials.

The *A*dventist *M*ulti-*e*thnic *N*utrition (AMEN) Study is a new pilot study from Loma Linda University involving Hispanic/Latino Seventh-day Adventist adults for the purpose of studying the association between their cultural tailoring of a plant-based diet and selected health outcomes. To date, we have observed beneficial associations between health outcomes and culturally-tailored plant-based diet choices in Black Adventists (24, 25) and in Asian Adventists (20), respectively. The aim of this report is to test the hypothesis that the culturally-tailored plant-based dietary pattern practiced by Hispanic/Latino Seventh-day Adventists is associated with lower levels of adiposity and adiposity-related biomarkers. Findings from this work can potentially inform the cultural tailoring of plant-based diet choices for dietary interventions in the broader population of US Hispanic/Latinos.

## Methods

### Study Population

The AMEN study is a cross-sectional investigation of diet and cardio-metabolic markers conducted during 2013–2016 in churches within a 20 mile radius of Loma Linda University. Our target congregations were predominantly of Hispanic/Latino background. The inclusion criteria included: (1) age 18 years or older, (2) baptized into the Seventh-day Adventist church, (3) self-identify as Hispanic, and (4) no self-report of dementia, pregnancy, or breastfeeding.

Recruitment efforts were initiated by assembling a community advisory board of influential individuals from the Hispanic and Asian Adventist churches in Southern California. Based on the advice of this board, we selected five Hispanic congregations. An additional criterion was that the congregations be meeting in facilities that could accommodate a weekend health clinic. Recruitment from these congregations was done by: (1) linking recruitment to informative health and lifestyle presentations done by Loma Linda University lifestyle medicine faculty, (2) linking recruitment to health and wellness clinics done on the church premises over a weekend, and (3) notices on church bulletin boards, social media, and through e-mail campaigns.

Through these efforts, we recruited 141 subjects from the five congregations that had been invited to participate, of whom 101 subjects were eligible for enrollment. Due to missing data for pertinent diet and anthropometric variables, our analytic sample consisted of 74 subjects. All data collection forms were translated into English and Spanish, and a bi-lingual translator was available for assistance.

### Outcome Measures

Eligible subjects were enrolled if they attended a health assessment clinic that occurred either as part of a health fair at their church or by appointment at Loma Linda University. A 12 h fasting blood sample drawn by a certified phlebotomist and first void urine sample were collected from each participant. For this analysis, biospecimens were analyzed for a comprehensive metabolic panel, and also tests of inflammatory markers (interleukin-6, C reactive protein) using a previously described protocol by Jaceldo-Siegl et al. (26). Briefly, collection of blood was done after an overnight fast, and serum was separated from the cells within 30 min of collection, placed on wet ice, and then shipped or transported to the central study laboratory at Loma Linda, CA for further processing. Blood was aliquoted to 0.5 mL samples and immediately stored in liquid nitrogen until analysis. Concentrations of CRP and IL-6 were measured in duplicate samples using enzyme linked immunosorbent assay (ELISA) kits from R & D Systems (Minneapolis, MN, USA) for IL-6, Thermo Fisher Scientific (Waltham, MA, USA), and Assaypro (St. Charles, MO, USA) for CRP. For IL-6, the detectable limit was 0.039 pg/mL, and intra- and inter-assay CV were 6.9 and 7.2%; and for CRP, these were 100 pg/mL, 5.5 and 7.6%, respectively.

Blood pressure (BP), weight, height, body mass index (BMI), waist circumference (WC), and body composition were assessed on-site. Height was measured to the nearest quarter inch (0.64 cm) using the Seca 214 Portable Height Rod (Seca Corp, Hamburg, Germany). Weight (measured to the nearest half pound or 0.23 kg) and body composition were assessed using a body composition analyzer (Tanita, Model TBF-300A, Arlington Heights, IL, USA). Waist circumference was obtained using an anthropometric tape placed around the waist just above the hipbone.

Blood pressure and resting pulse were assessed each as the average of three measurements using the OMRON Digital Blood Pressure Monitor HEM-7471C (Omron Healthcare Inc., Vernon Hills, IL, USA). All subjects were subject to a standard protocol, including a 5 min quiet relaxation period prior to the first reading of BP and resting pulse, and 1 min rest between consecutive measurements.

### Classification of Vegetarian Diet

Dietary intake was assessed using two methods: (1) A single 24 h recall using standard methodology that has been discussed previously (27), (2) Survey items adapted from EPIC Oxford where subjects were also asked about their consumption (in the past 30 days) of four animal-based products (meat, fish, dairy, and eggs). Using the latter method to survey usual intake, participants were classified as non-vegetarian if they currently eat meat; pescatarian if they eat no meat but eat fish; lacto-ovo vegetarian if they eat dairy and eggs but not meat or fish; and strict vegetarian if they do not consume meat, eggs, fish and dairy. Due to the limited sample size, strict vegetarians, lacto-ovo vegetarians, and pescatarians were considered as following a plant-based diet and collapsed into a “vegetarian” group. Survey items measuring meat intake among Seventh-day Adventists have been validated against multiple 24 h recalls with correlations that exceed 0.80 (27).

### Assessment of Demographic, Religiosity, and Culture-Specific Data

We obtained demographic, spoken language, nativity and religiosity data by questionnaire. Religiosity was assessed by asking the question, “How religious are you?” where we defined “religious” as having “to do with a personal experience and not just a behavior, like going to church.” Responses were elicited using a 10-point semantic differential scale where 1 represents not religious at all and 10 the most religious.

### Statistics

Descriptive analyses comparing vegetarians and non-vegetarians on selected subject characteristics were conducted using *t*-tests for continuous, and χ^2^ or Fisher test for categorical variables. Continuous outcome variables (anthropometric, body composition, pulse, and blood pressure measures) were compared in vegetarians and non-vegetarians using generalized linear models adjusted for age, sex, and education. Ninety-five percent confidence intervals for the difference between these adjusted means were computed using the standard errors of the regression coefficients in the models.

## Results

In Table 1, we examined demographics characteristics of Hispanic/Latino subjects in the AMEN study. We found that vegetarians tended to be older (*p* = 0.006) and more educated (*p* = 0.02) as compared to non-vegetarians (Table 1). There was no significant difference in religiosity between the vegetarians and non-vegetarians.

**Table 1:** Selected characteristics among Hispanic/Latino vegetarians and non-vegetarians in the Adventist Multi-ethnic Nutrition (AMEN) Study.

		**Vegetarian (*****n*** **= 23)**		**Non-vegetarian (*****n*** **= 51)**		***p*-value**
Age (y)^a^	Mean	54.3		47.6		0.03
	SD	14.1		10.6		
Religiosity	Mean	8.22		7.78		0.22
	SD	1.24		1.46		
		***n***	**%**	***n***	**%**	
**Sex^a^**						0.15
	Male	4	17.4	17	34.0	
	Female	19	82.6	33	66.0	
**Income^a^**						
	< $50,000	12	57.1	38	77.6	0.08
	≥$51,000	9	42.9	11	22.4	
**Education**						
	High school or Less	6	26.1	28	54.9	0.02
	College or more	17	73.9	23	45.1	
**Marital status**						
	Married	17	73.9	39	76.5	0.81
	Not Married	6	26.1	12	23.5	
**Language**						
	English	2	8.7	7	13.7	0.31
	Spanish	19	82.6	43	84.3	
	Other	2	8.7	1	2.0	
**Nativity**						
	U.S. Born	3	13.0	4	7.8	0.67
	Foreign Born	20	87.0	47	92.2	

a *% Missing data: 1% Age, 1% Sex, 2.08% Income*.

### Vegetarian Diet and Adiposity

In linear regression models (Table 2), we tested the association between measures of adiposity (BMI, waist circumference (WC), fat mass, and percent body fat) as outcomes and vegetarian diet status as a main exposure. We found that BMI was lower among the vegetarians than the non-vegetarians (24.5 kg/m^2^ vs. 27.9 kg/m^2^, *p* = 0.006) after adjusting for age, sex, and education. Vegetarians also had significantly lower waist circumference (34.8 in vs. 37.5 in), fat mass (18.3 kg vs. 23.9 kg), and percent body fat (28.4% vs. 32%) as compared to non-vegetarians. Pulse rate, systolic and diastolic blood pressure values among vegetarians were not significantly different from those of non-vegetarians.

**Table 2:** Comparison of obesity measures, body composition, pulse, and blood pressure in vegetarians and non-vegetarians in the AMEN Study.

	**Adjusted for age, sex, and education**		
	**Mean**	**Difference (95% CI)**	***p*-value**
**BODY MASS INDEX (kg/m**^**2**^**)**			
Non-veg^a^	27.9	3.3 (1.0, 5.7)	0.006
Veg	24.5		
**WAIST CIRCUMFERENCE (in)**			
Non-veg	37.5	2.8 (0.6, 4.9)	0.01
Veg	34.8		
**FAT MASS (kg)**			
Non-veg	23.9	5.5 (1.6, 9.5)	0.007
Veg	18.3		
**PERCENT BODY FAT**			
Non-veg	32.0	3.6 (0.5, 6.7)	0.025
Veg	28.4		
**PULSE RATE**			
Non-veg	67.5	2.8 (−2.4, 8.1)	0.28
Veg	64.6		
**SYSTOLIC BLOOD PRESSURE**			
Non-veg	117.6	−3.0 (−11.1, 5.1)	0.46
Veg	120.6		
**DIASTOLIC BLOOD PRESSURE**			
Non-veg	77.1	1.1 (−3.6, 5.9)	0.64
Veg	75.9		

a *Non-veg, non-vegetarian; Veg, vegetarian (combining vegan, lacto-ovo, and pesco-vegetarian)*.

### Vegetarian Diet, Adiposity, and Inflammatory Markers

We found that log-transformed interleukin-6 (IL-6) was positively associated with the following adiposity outcomes: BMI (β = 1.64, *p* = 0.04), waist circumference (β = 1.32, *p* = 0.08), fat mass (β = 1.32, *p* = 0.05), and fat percent (β = 4.22, *p* = 0.004). Adding the main exposure for vegetarian status to the model did not appreciably reduce these associations. For log transformed C-reactive protein (CRP), positive associations were found for fat mass (β = 2.38, *p* = 0.03) and fat percent (β = 3.13, *p* = 0.004). Similarly, adding the main exposure did not change CRP associations with measures of adiposity. We note that the power to detect mediation was low, making type 1 errors more likely.

There were no significant differences between vegetarians and non-vegetarians for log-transformed CRP (β = −0.06562, *p* = 0.78) or log-transformed IL-6 (β = −0.19132, *p* = 0.29).

## Discussion

In the AMEN study, we found that in a sample of Seventh-day Adventist Hispanic/Latino adults, those following a vegetarian dietary pattern had a BMI that was lower (24.5 kg/m^2^ vs. 27.9 kg/m^2^, *p* = 0.006) and within federally-recommended limits as compared to non-vegetarians. These findings were confirmed by similar decreases in other measures of adiposity [fat mass (18.3 kg vs. 23.9 kg), and percent body fat (28.4% vs. 32%)] and abdominal adiposity [waist circumference (34.8 in vs. 37.5 in)].

These findings are concordant with findings from non-Hispanic white Adventists (18, 19, 22), Black/African American Adventists (22, 25), and Asian Adventists (20). For Black Adventists, Akbar et al. has reported a “cultural tailoring” of the vegetarian diet chosen whereby Southern and Caribbean influenced dietary patterns introduced a wide range of plant foods into the diet (24). In US-based Asian Indian Adventists, Singh reported the predominance of nuts as a major source of protein in vegetarians who were at lower BMI (20).

Our next step in the AMEN study was to investigate how the protective patterns of plant-based eating in Hispanic/Latino adults were influenced by cultural preference for specific plant foods. These data can potentially inform culturally-tailored plant-based dietary interventions for the broader population of US Hispanic/Latinos who have been experiencing a nutrition transition away from simpler, more traditional diets rich in whole plant foods.

### Plant-Based Eating in US Hispanic/Latinos

Our data indicates that more plant-based eating in US-based Hispanic/Latino Adventist adults was associated on average with adiposity indices falling within the federally-recommended limits. If this association holds true also for non-Adventist Hispanics/Latinos, how feasible and scalable is it to have Adventist and non-Adventist Hispanics/Latinos increase their plant-based dietary choices as a means of preventing the obesity and cardiometabolic diseases that are disproportionately prevalent in their community?

When considering this question it is noteworthy that data from Latin America indicate a rich cultural tradition of eating and preparing a diverse range of regional plant foods. For example, the Tarahumara Indians of Mexico traditionally adhered to a diet consisting of beans, corn, and squash and very little meat, and this pattern has been associated with lower risk of cardiometabolic disease (28) and can be significantly compromised by plying the Tarahumara with a typical U.S. diet (29). Studies from South America report that vegan and semi-vegetarian Peruvian and Brazilian subjects (following traditional cultural choices involving plant foods) do have lower rates of hypertension, dyslipidemia, and obesity as compared to omnivores (30). These small studies provide clues that increasing plant-based diet choices based on long-held cultural traditions of eating whole plant foods can potentially be an attractive target for high impact interventions. When designing dietary interventions we note that the strict vegetarian diet pattern is not commonly practiced by US Hispanic/Latinos (31), and further research is needed in this community to examine whether increasing plant food intake to the point of semi-vegetarian or pescatarian pattern can improve health outcomes.

### Plant-Based Eating, Pro-Inflammatory Cytokines, and Obesity in Hispanic/Latinos

Among the Hispanic/Latino adults of the AMEN study, we did find that measures of adiposity were associated with pro-inflammatory cytokines such as interleukin-6. Compared to non-vegetarian dietary patterns, vegetarian dietary patterns generate increased microbially-generated short chain fatty acids, which reduce systemic inflammation (e.g., IL-6), increase hormonal satiety-signaling (e.g., glucagon-like peptide-1) and reduce insulin resistance (32, 33). Among Hispanic/Latino subjects, recent studies suggest that IL-6 may be elevated from a young age, and the link across the lifespan to higher rates of cardiometabolic disease may be due to specific IL-6 polymorphisms in Latino youth (34). Interestingly, adding IL-6 to our models examining association with vegetarian diet exposure did not alter the protective effect of the diet on obesity.

### Limitations

The AMEN study has important limitations. The cross-sectional and observational design precludes inferring a causal effect from the association of diet with obesity and obesity-related disease. The purpose of our pilot study was to generate culturally-specific hypotheses for testing this association in longitudinal and experimental designs with novel culturally-tailored interventions.

The relatively small sample size limited our statistical power to test more complex food and nutrient models, and for testing interaction and mediation. We note, however, the significant and cross-validating associations between plant-based eating and measures of adiposity that persist despite the limited power to detect these associations.

Religiosity could also be a factor since we are studying a faith-based group. It is noteworthy, however, that we observed no difference in religiosity between the vegetarians and non-vegetarians we studied. Measurement error in dietary assessment is always present in observational work, but we note a decades-long track record by our research group validating diet and vegetarian diet survey measures in Adventists (16, 17, 35).

## Conclusions

Our findings from Hispanic/Latino Seventh-day Adventists indicate that plant-based diet patterns were associated with adiposity in the recommended range, and effects were consistent across multiple measures of adiposity and obesity-related inflammatory markers. Further studies of how Hispanics/Latinos have culturally tailored the Adventist plant-based eating pattern could potentially inform interventions for the broader population of Hispanics/Latinos.

## Ethics Statement

The study protocol was approved by the institutional review board of Loma Linda University and performed in accordance with the ethical standards as laid down in the 1975 Declaration of Helsinki as revised in 2000. All participants provided signed informed consent.

## Author Contributions

PS: designed the study, supervised data collection and analysis, wrote the final version of the manuscript and obtained funding; KJ-S: designed the study, supervised data collection and analysis, wrote a draft of the manuscript; NC: analyzed data; WS: analyzed data and edited manuscript; LL: data collection coordinator and preliminary analysis; KS: analyzed data and edited manuscript; DE: analyzed data; MJ, HF, DH-B, and WM: edited manuscript.

### Conflict of Interest Statement

The authors declare that the research was conducted in the absence of any commercial or financial relationships that could be construed as a potential conflict of interest.

## Abbreviations

AMEN: Adventist Multi-*e*thnic Nutrition Study
US: United States
T2D: Type 2 Diabetes
HbA1c: Hemoglobin A1c
WC: Waist circumference
BMI: Body mass index
BP: blood pressure
AHS-2: Adventist Health Study 2
Veg: Vegetarian
Non-veg: Non-vegetarian.
