# Corrigendum: Plant-Based Diets Are Associated With Lower Adiposity Levels Among Hispanic/Latino Adults in the Adventist Multi-Ethnic Nutrition (AMEN) Study

**DOI:** 10.3389/fnut.2019.00088

**Published:** 2019-06-12

**Authors:** Pramil N. Singh, Karen Jaceldo-Siegl, Wendy Shih, Nancy Collado, Lap T. Le, Krystal Silguero, Dennys Estevez, Michael Jordan, Hector Flores, David E. Hayes-Bautista, William J. McCarthy

**Affiliations:** ^1^Center for Nutrition, Healthy Lifestyles and Disease Prevention, School of Public Health, Loma Linda University, Loma Linda, CA, United States; ^2^Center for Health Research, School of Public Health, Loma Linda University, Loma Linda, CA, United States; ^3^Center for Hispanic Health, White Memorial Medical Center, Los Angeles, CA, United States; ^4^Department of Family Medicine, White Memorial Medical Center, Los Angeles, CA, United States; ^5^Center for Study of Latino Health and Culture, David Geffen School of Medicine, University of California at Los Angeles, Los Angeles, CA, United States; ^6^Health Policy and Management, School of Public Health, UCLA Jonsson Comprehensive Cancer Center, Los Angeles, CA, United States

**Keywords:** Hispanic/Latino, plant-based diet, vegetarian, obesity, Seventh-day Adventist

In the original article, there was a mistake in Table 2 as published. The waist circumference results were provided in cm and should in fact be in inches. The corrected Table 2 appears below.

**Table 2:** Comparison of obesity measures, body composition, pulse, and blood pressure in vegetarians and non-vegetarians in the AMEN Study.

	**Adjusted for age, sex, and education**		
	**Mean**	**Difference (95% CI)**	***p*-value**
**BODY MASS INDEX (kg/m**^**2**^**)**			
Non-veg^a^	27.9	3.3 (1.0, 5.7)	0.006
Veg	24.5		
**WAIST CIRCUMFERENCE (in)**			
Non-veg	37.5	2.8 (0.6, 4.9)	0.01
Veg	34.8		
**FAT MASS (kg)**			
Non-veg	23.9	5.5 (1.6, 9.5)	0.007
Veg	18.3		
**PERCENT BODY FAT**			
Non-veg	32.0	3.6 (0.5, 6.7)	0.025
Veg	28.4		
**PULSE RATE**			
Non-veg	67.5	2.8 (−2.4, 8.1)	0.28
Veg	64.6		
**SYSTOLIC BLOOD PRESSURE**			
Non-veg	117.6	−3.0 (−11.1, 5.1)	0.46
Veg	120.6		
**DIASTOLIC BLOOD PRESSURE**			
Non-veg	77.1	1.1 (−3.6, 5.9)	0.64
Veg	75.9		

a *Non-veg, non-vegetarian; Veg, vegetarian (combining vegan, lacto-ovo, and pesco-vegetarian)*.

Additionally, throughout the article the waist circumference was listed in centimeters and should in fact be in inches.

Corrections have therefore been made to the following section:

The **Abstract, paragraph four**:

“**Results:** Vegetarian diet patterns (Vegan, Lacto-ovo vegetarian, Pesco-vegetarian) were associated with significantly lower BMI (24.5 kg/m^2^ vs. 27.9 kg/m^2^, *p* = 0.006), waist circumference (34.8 in vs. 37.5 in, *p* = 0.01), and fat mass (18.3 kg vs. 23.9 kg, *p* = 0.007), as compared to non-vegetarians. Adiposity was positively associated with pro-inflammatory cytokines (Interleukin-6) in this sample, but adjusting for this effect did not alter the associations with vegetarian diet.”

The **Results**, subsection **Vegetarian Diet and Adiposity**:

“In linear regression models (Table 2), we tested the association between measures of adiposity (BMI, waist circumference (WC), fat mass, and percent body fat) as outcomes and vegetarian diet status as a main exposure. We found that BMI was lower among the vegetarians than the non-vegetarians (24.5 kg/m^2^ vs. 27.9 kg/m^2^, *p* = 0.006) after adjusting for age, sex, and education. Vegetarians also had significantly lower waist circumference (34.8 in vs. 37.5 in), fat mass (18.3 kg vs. 23.9 kg), and percent body fat (28.4% vs. 32%) as compared to non-vegetarians. Pulse rate, systolic and diastolic blood pressure values among vegetarians were not significantly different from those of non-vegetarians.”

The **Discussion, paragraph one**:

“In the AMEN study, we found that in a sample of Seventh-day Adventist Hispanic/Latino adults, those following a vegetarian dietary pattern had a BMI that was lower (24.5 kg/m^2^ vs. 27.9 kg/m^2^, *p* = 0.006) and within federally-recommended limits as compared to non-vegetarians. These findings were confirmed by similar decreases in other measures of adiposity [fat mass (18.3 kg vs. 23.9 kg), and percent body fat (28.4% vs. 32%)] and abdominal adiposity [waist circumference (34.8 in vs. 37.5 in)].”

The authors apologize for these errors and state that they do not change the scientific conclusions of the article in any way. The original article has been updated.

